# Integrated Risk Stratification Using Comorbidity Burden, Frailty, and Systemic Inflammation to Predict 30-Day Mortality in Older Emergency Department Patients: A Prospective Cohort Study

**DOI:** 10.3390/jcm15186954

**Published:** 2026-09-08

**Authors:** Kamil Konur, Nurullah Parca, Bunyamin Onur Harmanci, Gulfidan Atan, Kadir Can Kamaci, Metin Yildiztac, Erol Karavar, Ismail Atas, Mumin Murat Yazici, Zeynep Irmak Kaya, Abdullah Bora Ozkara, Hatice Beyazal Polat, Ozlem Bilir

**Affiliations:** 1 Department of Internal Medicine, Faculty of Medicine, Recep Tayyip Erdogan University, 53020 Rize, Turkey; glfdnatan@gmail.com (G.A.); k.can_99@hotmail.com (K.C.K.); mtnyldztc@gmail.com (M.Y.); erolkaravar@gmail.com (E.K.); hatice.beyazalpolat@erdogan.edu.tr (H.B.P.); 2 Department of Emergency Medicine, Recep Tayyip Erdogan University Training and Research Hospital, 53020 Rize, Turkey; nrllhprca@gmail.com (N.P.); gayiboglu01@gmail.com (B.O.H.); drismailatas@gmail.com (I.A.); mmuratyazici53@gmail.com (M.M.Y.); 3 Department of Internal Medicine, Health Sciences University Eskisehir Health Application and Research Center, Eskisehir City Hospital, 26080 Eskisehir, Turkey; dr.zeynepirmak@gmail.com; 4 Faculty of Health Sciences, Karadeniz Technical University, 61080 Trabzon, Turkey; bora.ozkara@ktu.edu.tr; 5 Department of Emergency Medicine, Faculty of Medicine, Recep Tayyip Erdogan University, 53020 Rize, Turkey; drozlembilir@gmail.com

**Keywords:** Charlson comorbidity index, clinical frailty scale, systemic immune-inflammation index, geriatric emergency medicine, risk stratification

## Abstract

**Background/Objectives**: Estimating mortality risk in older adults presenting to the emergency department (ED) is challenging because risk is shaped by comorbidity burden, frailty, and systemic inflammation. We examined the individual and combined prognostic contributions of the Age-adjusted Charlson Comorbidity Index (CCI), the Clinical Frailty Scale (CFS), and the Systemic Immune-Inflammation Index (SII) to 30-day mortality. **Methods**: This prospective observational cohort included 326 adults aged ≥65 years evaluated in a tertiary ED from September 2025 through April 2026. CCI, CFS, and SII were assessed at admission, with SII analyzed after logarithmic transformation [ln(SII)]. Associations with 30-day mortality were examined using logistic regression. Discriminative performance was evaluated by receiver operating characteristic analysis, and the integrated model underwent bootstrap internal validation and calibration assessment. **Results**: Thirty-day mortality occurred in 60 patients (18.4%). CCI, CFS, and ln(SII) were each independently associated with mortality in the integrated multivariable model (all *p* < 0.05). Individual AUCs were 0.741 for CCI, 0.703 for CFS, and 0.662 for ln(SII), with CCI showing the highest value. The integrated model achieved the highest observed discrimination (AUC 0.793; optimism-corrected AUC 0.783) and showed acceptable internal calibration (Brier score 0.127; bootstrap-corrected calibration intercept 0.003; calibration slope 0.957). **Conclusions**: Comorbidity burden, frailty, and systemic inflammation each provided independent prognostic information for 30-day mortality in older ED patients. Although CCI was the strongest individual predictor, the integrated model achieved the highest observed discrimination. However, its incremental benefit over some two-predictor models was modest and not statistically significant.

## 1. Introduction

Population aging is progressively changing the demographic profile of patients presenting to EDs. Older adults represent an increasing share of ED presentations and face a greater risk of morbidity and mortality than younger individuals [1]. This increased vulnerability reflects the combined influence of declining physiological reserve, coexisting chronic conditions, and progressive functional impairment rather than the effect of any single disease. Together, these factors render older adults considerably more susceptible to adverse outcomes following acute illness or trauma [2].

The CCI is commonly used in clinical practice to quantify comorbidity burden [3]. By identifying and weighting coexisting medical conditions, the CCI summarizes overall comorbidity burden into a single score and has been extensively validated for predicting survival and mortality across diverse clinical settings [4,5]. However, comorbidity burden alone does not fully capture the biological vulnerability of older adults. This limitation has increased attention to frailty, a multidimensional state in which physiological reserve and the capacity to withstand health stressors are diminished, independently of chronological age. Frailty has been associated with a range of adverse outcomes, including postoperative complications, falls, delirium, prolonged hospitalization, and mortality [6,7].

Aging is also accompanied by persistent low-grade systemic inflammatory activity, commonly described as inflammaging [8]. The SII combines peripheral neutrophil, platelet, and lymphocyte counts into a readily obtainable marker of systemic immune-inflammatory activity based on routine laboratory data [9,10]. These three measures were selected because they represent complementary and readily assessable dimensions of vulnerability in older adults presenting to the emergency department: CCI reflects cumulative chronic disease burden, CFS captures baseline functional reserve and frailty, and SII provides a routinely available measure of systemic immune-inflammatory activity. Although other geriatric domains, including nutritional and cognitive status, are also clinically important, the present study was designed to evaluate a parsimonious combination of measures that can be obtained rapidly from clinical history, bedside assessment, and routine laboratory testing in the ED.

Frailty assessment, particularly using the CFS, has been widely studied for prognostic evaluation in older ED populations [11], and combinations of frailty and comorbidity burden have also been evaluated for short-term mortality prediction in older patients [12]. However, studies directly comparing the prognostic contributions of CCI, CFS, and SII within a single model for short-term mortality in older adults presenting to the ED remain scarce.

Reliable risk estimation in older ED patients may facilitate clinical decision-making, appropriate resource allocation, and patient management [13]. Integrating assessments of comorbidity burden, frailty, and systemic inflammation may provide a more comprehensive prognostic evaluation than relying on a single measure alone.

This study aimed to examine the associations of CCI-defined comorbidity burden, CFS-defined frailty, and SII-defined systemic inflammation with 30-day mortality in older adults presenting to the ED. In addition, we compared the prognostic performance of these three parameters for predicting 30-day mortality.

## 2. Materials and Methods

### 2.1. Study Design and Setting

We conducted a prospective observational cohort study in the Emergency Department of Recep Tayyip Erdoğan University Training and Research Hospital from September 2025 through April 2026. Patient recruitment was completed in April 2026, and the final 30-day follow-up was completed in May 2026. Ethical approval was obtained from the Recep Tayyip Erdoğan University Non-Interventional Clinical Research Ethics Committee (Approval No. 2025/366; 21 August 2025), and the study was conducted in accordance with the Declaration of Helsinki. Written informed consent was obtained from all participants or their legally authorized representatives before enrollment.

### 2.2. Study Population

Patients aged 65 years or older presenting to the emergency department were prospectively recruited using non-consecutive convenience sampling during periods when study personnel were available. Eligibility for the analytic cohort required written informed consent, complete clinical and laboratory information, complete follow-up data, and ascertainment of 30-day mortality status.

Patients with acute infection or autoimmune disease, patients transferred to another healthcare institution before completion of follow-up, and those with missing or incomplete clinical data were excluded. Acute infection and autoimmune disease were excluded because these conditions may substantially alter the neutrophil, lymphocyte, and platelet counts used to calculate SII, potentially causing SII to predominantly reflect an acute or disease-related inflammatory perturbation.

### 2.3. Sample Size

The available cohort comprised 326 participants, of whom 60 experienced 30-day mortality. The primary multivariable model was intentionally limited to the three principal predictors of interest—CCI, CFS, and ln(SII)—to maintain a parsimonious model in relation to the available number of outcome events. No formal prediction-model sample-size calculation was used to establish the adequacy of the cohort for model development.

### 2.4. Data Collection

Recorded baseline demographic and anthropometric data included age, sex, height, body weight, and waist circumference. Medical history was reviewed for hypertension, diabetes mellitus, coronary artery disease, congestive heart failure, chronic kidney disease, cerebrovascular disease, liver disease, peptic ulcer disease, peripheral vascular disease, chronic pulmonary disease, and malignancy. Smoking status, physical activity level, and use of immunosuppressive medications were recorded.

Laboratory measurements from the initial ED blood samples included white blood cell, neutrophil, lymphocyte, monocyte, hemoglobin, platelet, glucose, urea, creatinine, estimated glomerular filtration rate (eGFR), albumin, alanine aminotransferase (ALT), aspartate aminotransferase (AST), and C-reactive protein (CRP).

All patients were followed prospectively for 30 days. Mortality status was determined through review of hospital electronic medical records and, when necessary, by telephone contact with the patients or their relatives. If the initial telephone attempt was unsuccessful, a second contact attempt was made. Complete 30-day follow-up data were available for all 326 included patients, and no patients were lost to follow-up.

Baseline demographic and clinical data were collected at the time of emergency department admission. Frailty assessment was performed during the same ED evaluation and reflected the patient’s baseline functional status before the acute illness.

### 2.5. Study Variables and Outcome Measures

The three principal prognostic domains were comorbidity burden, frailty, and systemic inflammation, represented by the CCI, the CFS, and the SII, respectively. Thirty-day all-cause mortality was the primary study outcome.

CCI: The age-adjusted CCI was calculated from comorbid conditions documented in the medical record, with the appropriate age-related points added to the total score [3,14]. The CCI and its age-adjusted version have been extensively validated for mortality prediction across clinical populations.

CFS: At ED presentation, frailty was rated with the validated 9-point Rockwood CFS [15,16]. The 9-point CFS has also demonstrated good validity and reliability in the Turkish older population [16], and has shown predictive validity and inter-rater reliability in older emergency department populations [17]. No fixed retrospective time window was specified; the assessment was intended to reflect the patient’s usual baseline functional status before the acute illness. Information regarding usual mobility, level of independence, and need for assistance was obtained from the patient and/or a relative. For patients who were unable to communicate reliably because of conditions such as delirium, cognitive impairment, or critical illness, the CFS assessment was based on collateral information obtained from a relative regarding the patient’s usual functional status before the acute illness. When information was available from both sources, it was considered jointly; patients were excluded if clinically relevant discrepancies between patient and proxy information could not be resolved. Although the assessors did not receive formal CFS training, assessments were performed using the same standard 9-point CFS descriptors. No formal inter-rater reliability assessment was performed.

SII: SII was derived from the admission complete blood count by combining platelet, neutrophil, and lymphocyte counts according to the established formula [18]:

SII = (Platelet count × Neutrophil count)/Lymphocyte count

### 2.6. Statistical Analysis

All statistical analyses were conducted in jamovi version 1.6 (The jamovi Project, Sydney, Australia). Distributional normality of continuous variables was examined with the Kolmogorov–Smirnov test. Normally distributed continuous data are summarized as mean (standard deviation [SD]), non-normally distributed data as median (interquartile range [IQR]), and categorical data as counts and percentages.

The final analytic cohort consisted of 326 participants with complete clinical, laboratory, and follow-up data, including complete data for CCI, CFS, SII, and 30-day mortality status. Therefore, no imputation of missing data was required or performed.

Between-group comparisons used Student’s *t*-test for normally distributed variables and the Mann–Whitney U test otherwise. Categorical data were analyzed with the chi-square test, with Fisher’s exact test applied when expected cell counts were too small for the chi-square approximation.

Separate univariable logistic regression models were fitted for CCI, CFS, and SII with 30-day mortality as the outcome. Because SII showed a right-skewed distribution, it was transformed using the natural logarithm [ln(SII)] before regression modelling. All SII values were greater than zero; therefore, addition of a constant before logarithmic transformation was not required. Effect estimates are reported as odds ratios (ORs) with 95% confidence intervals (CIs).

The primary multivariable logistic model simultaneously included CCI, CFS, and ln(SII), so that each effect estimate was adjusted for the other two predictors. Chronological age was not entered separately because the CCI used in this study was age-adjusted and already incorporated age-related points into the total score. Malignancy was likewise not entered separately into the primary model because it is a weighted component of the CCI; the robustness of the findings with respect to malignancy was evaluated in a separate sensitivity analysis excluding patients with malignancy. An additional sensitivity analysis was performed by adding sex to the primary three-predictor model. A further sensitivity analysis was performed by additionally adjusting the primary model for immunosuppressive medication use to assess whether the association between ln(SII) and 30-day mortality remained independent of this potential source of confounding. The regression coefficients and intercept from the final integrated model were used to construct the individual 30-day mortality prediction equation, which is reported in the Results section. Model discrimination was quantified from receiver operating characteristic (ROC) curves using the area under the curve (AUC) and its 95% confidence interval. Differences between AUCs were tested pairwise with the DeLong method. Internal validation of the integrated model was based on 1000 bootstrap resamples. Model calibration was assessed using the Brier score, a calibration plot, calibration-in-the-large (calibration intercept; ideal value, 0), and the calibration slope (ideal value, 1). Optimism-corrected estimates of the calibration intercept and slope were obtained using 1000 bootstrap resamples. The Hosmer–Lemeshow goodness-of-fit test was retained as a supplementary calibration assessment. Statistical significance was defined as a two-sided *p* value < 0.05.

## 3. Results

The study included 326 patients, of whom 60 (18.4%) died within 30 days and 266 (81.6%) survived.

### 3.1. Demographic and Clinical Characteristics of the Study Population

Male sex was more frequent among non-survivors than survivors (71.7% vs. 51.9%, *p* = 0.005). Age and height did not differ significantly between the mortality and survivor groups (*p* = 0.328 and *p* = 0.075, respectively). Non-survivors had lower body weight (75 vs. 80 kg, *p* = 0.002) and smaller waist circumference (97 ± 20 vs. 108 ± 17 cm, *p* < 0.001) than survivors.

Of the comorbid conditions examined, cerebrovascular disease (23.3% vs. 9.8%, *p* = 0.004) and malignancy (48.3% vs. 12.4%, *p* < 0.001) were more frequent among non-survivors. The prevalence of hypertension, diabetes mellitus, coronary artery disease, congestive heart failure, chronic kidney disease, liver disease, peptic ulcer disease, peripheral vascular disease, and chronic pulmonary disease was similar between groups. Smoking status and physical activity level were also comparable between the two groups. Immunosuppressive medication use was more common among non-survivors (28.3% vs. 8.3%, *p* < 0.001) (Table 1).

### 3.2. Laboratory Parameters and Values of SII, CFS, and CCI

Compared with survivors, non-survivors had higher white blood cell, neutrophil, platelet, and C-reactive protein values and lower hemoglobin and albumin levels. Lymphocyte and monocyte counts, glucose, urea, creatinine, eGFR, ALT, and AST were not significantly different between groups.

Median SII, CFS, and CCI values were higher among non-survivors than survivors (1520 vs. 794, 5 vs. 4, and 7 vs. 5, respectively; all *p* < 0.001) (Table 2).

### 3.3. Univariable and Multivariable Logistic Regression Analyses

Univariable models showed significant associations of CCI, CFS, and ln(SII) with 30-day mortality. The odds of 30-day mortality increased by 43.5% per one-point increase in CCI (OR 1.435, 95% CI 1.263–1.631; *p* < 0.001) and by 54.6% per one-point increase in CFS (OR 1.546, 95% CI 1.301–1.837; *p* < 0.001). ln(SII) was likewise positively associated with 30-day mortality (OR 1.783, 95% CI 1.330–2.389; *p* < 0.001).

When CCI, CFS, and ln(SII) were entered simultaneously into the integrated model, each retained an independent association with 30-day mortality. After mutual adjustment, each additional CCI point corresponded to a 32.0% higher odds of mortality (adjusted OR 1.320, 95% CI 1.145–1.520; *p* < 0.001), whereas each additional CFS point corresponded to a 24.4% higher odds (adjusted OR 1.244, 95% CI 1.022–1.515; *p* = 0.030). ln(SII) also remained independently associated with mortality (adjusted OR 1.500, 95% CI 1.096–2.053; *p* = 0.011) (Table 3).

To allow individual risk estimation and full reproducibility of the model, the fitted logistic regression equation was:

logit(p) = −7.077995 + 0.277325 × CCI + 0.218568 × CFS + 0.405593 × ln(SII),

where p represents the predicted probability of 30-day mortality. The individual predicted probability can therefore be calculated as p = 1/[1 + exp(−logit(p))].

In the sensitivity analysis excluding patients with malignancy, CCI remained independently associated with 30-day mortality (adjusted OR 1.313, 95% CI 1.061–1.626; *p* = 0.012), whereas CFS was no longer statistically significant (adjusted OR 1.241, 95% CI 0.956–1.611; *p* = 0.105). ln(SII) remained associated with mortality, although the estimate was close to the conventional threshold for statistical significance (adjusted OR 1.485, 95% CI 1.004–2.198; *p* = 0.048) (Supplementary Table S1).

In an additional sensitivity analysis adjusting the primary model for sex, CCI (adjusted OR 1.303, 95% CI 1.127–1.507; *p* < 0.001), CFS (adjusted OR 1.329, 95% CI 1.080–1.635; *p* = 0.007), and ln(SII) (adjusted OR 1.423, 95% CI 1.033–1.961; *p* = 0.031) all remained independently associated with 30-day mortality (Supplementary Table S2).

In a further sensitivity analysis additionally adjusting for immunosuppressive medication use, ln(SII) remained independently associated with 30-day mortality (adjusted OR 1.492, 95% CI 1.087–2.048; *p* = 0.013). CCI (adjusted OR 1.271, 95% CI 1.101–1.469; *p* = 0.001) and CFS (adjusted OR 1.250, 95% CI 1.023–1.529; *p* = 0.029) also remained independently associated with mortality (Supplementary Table S3).

### 3.4. Diagnostic Performance of SII, CFS, and CCI for Predicting 30-Day Mortality

ROC analysis demonstrated that the integrated model incorporating CCI, CFS, and ln(SII) achieved the highest discriminative performance for predicting 30-day mortality (AUC 0.793, bootstrap 95% CI 0.741–0.851), followed by the CCI + ln(SII) model (AUC 0.777) and the CCI + CFS model (AUC 0.772). For the individual predictors, the AUCs were 0.741 for CCI, 0.703 for CFS, and 0.662 for ln(SII).

**Figure 1:**
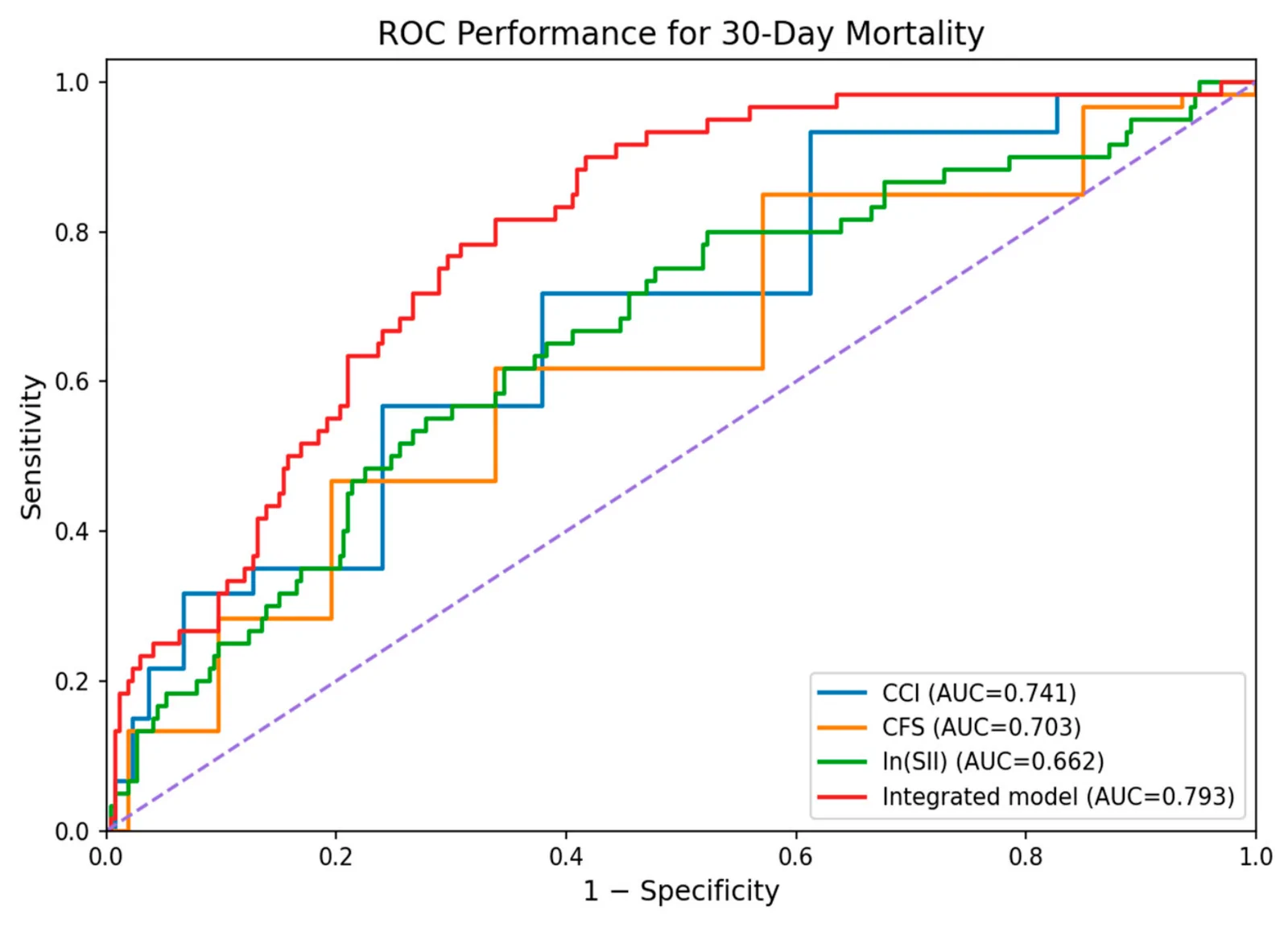
Receiver Operating Characteristic (ROC) curves of individual predictors and the integrated model for predicting 30-day mortality.

Pairwise DeLong comparisons demonstrated that the integrated model significantly outperformed CCI (ΔAUC 0.052, *p* = 0.015), CFS (ΔAUC 0.090, *p* < 0.001), and ln(SII) (ΔAUC 0.131, *p* = 0.001). In contrast, its AUC did not differ significantly from that of CCI + CFS (ΔAUC 0.021, *p* = 0.160) or CCI + ln(SII) (ΔAUC 0.016, *p* = 0.245). Detailed performance metrics are presented in Table 4, and the corresponding ROC curves are shown in Figure 1.

### 3.5. Internal Validation and Calibration of the Integrated Model

Bootstrap internal validation with 1000 resamples reduced the apparent AUC only slightly, from 0.793 to an optimism-corrected value of 0.783, consistent with limited optimism.

**Figure 2:**
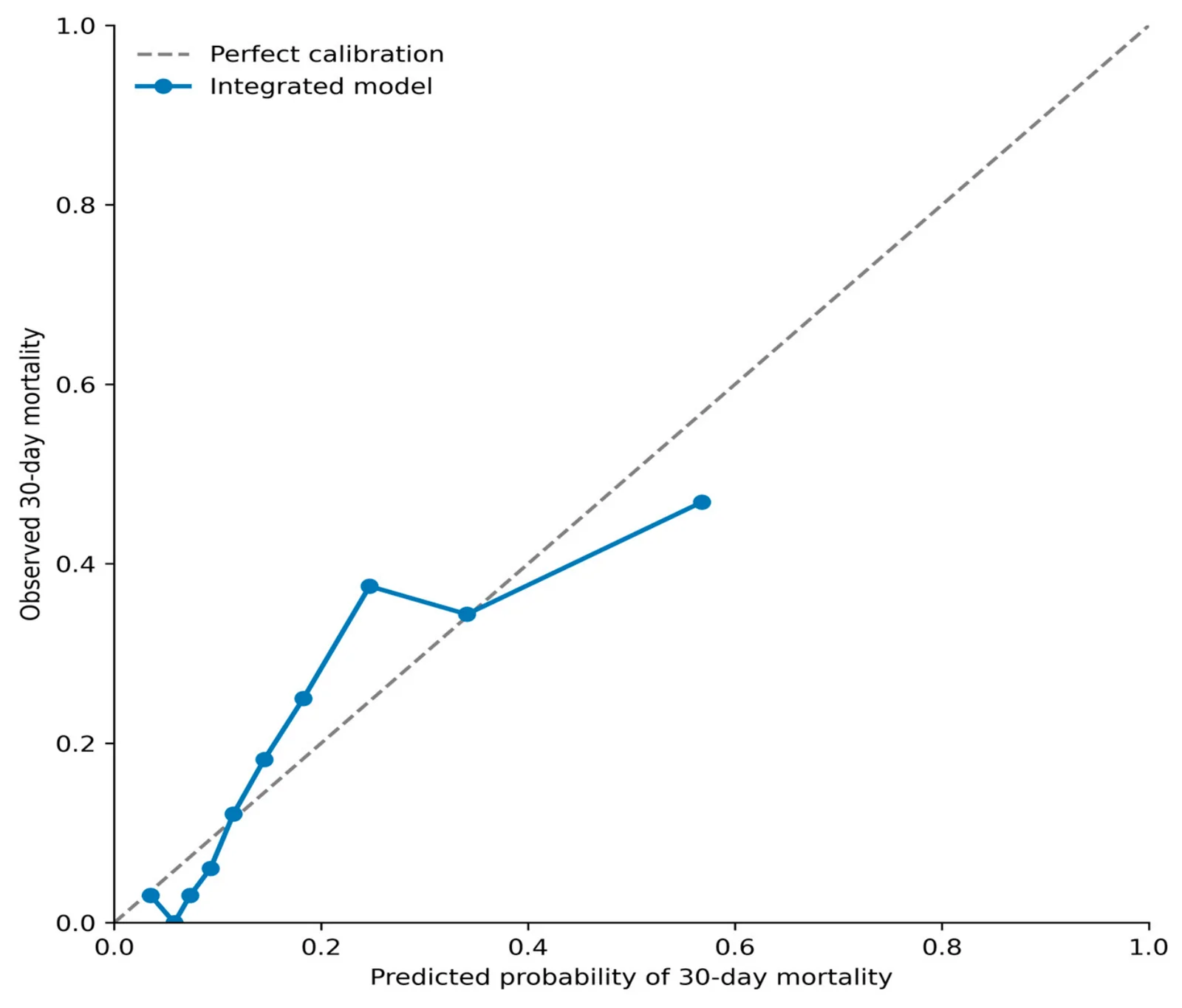
Calibration plot of the integrated model for 30-day mortality. The dashed 45° line denotes ideal calibration. Circles connected by the solid line show observed 30-day mortality across deciles of model-predicted risk for the model incorporating CCI, CFS, and ln(SII).

Model calibration was further assessed using the calibration intercept and slope in addition to the Brier score and graphical assessment. Following bootstrap correction, the calibration-in-the-large intercept was 0.003 and the calibration slope was 0.957, both close to their ideal values of 0 and 1, respectively. The Brier score was 0.127. The Hosmer–Lemeshow goodness-of-fit test yielded a *p* value of 0.358; however, this test was considered supplementary to the graphical and quantitative calibration measures. The calibration plot showed generally reasonable agreement between predicted and observed risks, although some deviation was observed across the intermediate risk range (Figure 2).

## 4. Discussion

This prospective cohort examined whether comorbidity burden, frailty, and systemic inflammation were associated with 30-day mortality among older adults evaluated in the emergency department. A key finding was that CCI, CFS, and log-transformed SII retained independent associations with mortality when considered simultaneously in the integrated model. This pattern is consistent with previous evidence linking greater comorbidity burden to mortality risk in older populations [19,20,21]. The highest observed discrimination was obtained with the integrated model (AUC 0.793), which also showed acceptable calibration and little optimism after bootstrap validation. Its discrimination was significantly greater than that of CCI alone; however, as discussed below, the incremental improvement over some two-predictor combinations was not statistically significant. Collectively, these findings suggest that comorbidity burden, frailty, and systemic inflammation represent complementary dimensions of vulnerability, while the extent of the incremental predictive benefit obtained by combining all three domains should be interpreted cautiously.

Our demographic findings are noteworthy. Non-survivors were more frequently male and had lower body weight and smaller waist circumference than survivors. These findings may reflect differences in underlying nutritional status, body composition, chronic disease burden, or acute illness severity. These findings should be interpreted cautiously because body weight and waist circumference were measured only at ED presentation, and longitudinal weight history or direct measures of muscle mass were not available. Chronological age did not differ significantly between survivors and non-survivors. Because the CCI used in our analyses was age-adjusted, age-related points were already incorporated into the CCI score; therefore, chronological age was not entered separately into the primary multivariable model. Lower albumin levels were also observed in non-survivors, a finding that may reflect both nutritional factors and the acute-phase response [22,23].

Among the individual comorbid conditions examined, significant associations with 30-day mortality were observed only for cerebrovascular disease and malignancy. Similarly, previous studies have shown that a greater burden of multiple chronic conditions is associated with increased mortality in older populations [24,25]. The absence of significant associations for several common conditions, including hypertension, diabetes mellitus, and coronary artery disease, suggests that overall comorbidity burden may be more prognostically informative than any single diagnosis. This interpretation is consistent with the strong prognostic performance of CCI observed in our study. CCI also retained an independent association with mortality after accounting for frailty and systemic inflammation, indicating that comorbidity burden contributed prognostic information beyond the other two domains.

Among the individual predictors evaluated, CCI demonstrated the strongest discriminative performance for 30-day mortality (AUC 0.741), compared with CFS (AUC 0.703) and ln(SII) (AUC 0.662). The stronger discriminative performance of CCI should, however, be interpreted in the context of the markedly higher prevalence of malignancy among non-survivors, as malignancy is a weighted component of the CCI and may therefore have contributed to its prognostic performance in this cohort. Accordingly, higher observed discriminative performance of CCI should not be interpreted as indicating that comorbidity burden uniformly dominates the other prognostic domains. Nevertheless, CFS and ln(SII) remained independently associated with mortality in the integrated model, indicating that frailty and systemic inflammation provided additional prognostic information beyond comorbidity burden alone. Previous studies have likewise demonstrated prognostic value of the CCI for both short- and long-term mortality [20,21]. The higher observed discriminative performance of CCI may reflect its ability to capture the cumulative burden of chronic diseases, which may influence physiological reserve and resilience to acute illness and thereby contribute to short-term mortality risk. In addition, the CCI is simple, objective, and can be readily calculated from routinely available clinical records, making it a practical tool for risk assessment in the emergency department. However, incorporating frailty and systemic inflammation with CCI resulted in the highest observed discrimination, although this finding should be interpreted in light of the modest incremental improvement and the lack of statistically significant differences compared with some two-predictor models.

CFS was also significantly associated with 30-day mortality, demonstrating moderate discriminative performance in the ROC analysis (AUC, 0.703). Similar associations between CFS scores, mortality, and other adverse outcomes have been reported in older ED populations [17,26,27]. Frailty may therefore capture prognostic information related to physiological reserve and tolerance of acute stress that is not fully represented by comorbidity burden. Although lower physical activity was more frequent among non-survivors than survivors (90.0% vs. 79.7%), this difference did not reach statistical significance (*p* = 0.063). Given the close relationship between physical activity and frailty, this descriptive finding may reflect reduced functional reserve; however, it should be interpreted cautiously. Because CFS requires no laboratory testing and can be assessed rapidly at the bedside, it may be particularly suitable for use in the ED. Within the primary integrated model, the CFS association persisted after accounting for CCI and ln(SII), supporting an additional contribution from frailty.

The findings regarding systemic inflammation were also noteworthy. Log-transformed SII retained an independent association with mortality after CCI and CFS were included in the model, suggesting additional prognostic information from the inflammatory domain. Logarithmic transformation of SII improved the interpretability of the regression model by accounting for the highly skewed distribution and large numerical scale of the original index. Despite having the lowest individual AUC, ln(SII) remained independently informative in the combined model, indicating that inflammatory status may capture risk not fully reflected by CCI or CFS. Because SII was measured during the initial ED evaluation, its components may reflect not only underlying systemic inflammatory status but also the acute physiological and inflammatory response associated with the presenting illness. Thus, the prognostic association observed for SII likely represents the combined influence of baseline inflammatory vulnerability and acute illness-related changes. The higher CRP levels observed among non-survivors provide additional evidence of greater inflammatory activity in this group. Previous studies have likewise reported heterogeneous findings between SII and mortality or other adverse clinical outcomes in older adults, although its prognostic performance appears to vary according to the clinical setting and patient population [28,29]. In the sensitivity analysis excluding patients with malignancy, CCI remained independently associated with mortality, whereas the association with CFS was attenuated and no longer statistically significant, and the association with ln(SII) remained only marginally significant. These findings indicate that the relative contributions of the three prognostic domains were sensitive to cohort composition. The exclusion of patients with malignancy also substantially reduced the number of mortality events, which may have contributed to wider confidence intervals and reduced statistical precision. Nevertheless, the attenuation of the CFS and SII associations suggests that the independent contributions of frailty and systemic inflammation should be interpreted cautiously and require confirmation in larger external cohorts.

Previous studies have established the prognostic relevance of frailty and broader geriatric risk assessment in older emergency department populations [13,26], while systemic inflammatory indices have also been associated with adverse outcomes in older adults [28,29]. Our study extends this literature by evaluating comorbidity burden, frailty, and systemic inflammation concurrently within the same cohort. CCI, CFS, and ln(SII) each remained independently associated with 30-day mortality in the integrated model. However, although the three-predictor model achieved the highest observed AUC, its discrimination was not significantly greater than that of the CCI + CFS or CCI + ln(SII) models. Thus, our findings support the complementary prognostic contributions of these domains but do not establish unequivocal superiority of the three-predictor model over simpler combinations. Further external validation is needed to determine the incremental clinical value of combining all three domains.

Taken individually, all three predictors showed only moderate discrimination, limiting their suitability as standalone risk-stratification tools. The integrated three-predictor model achieved the highest observed AUC (0.793). Compared with CCI alone, the increase in discrimination was modest (ΔAUC = 0.052) but statistically significant. However, the integrated model did not significantly outperform the CCI + CFS or CCI + ln(SII) models (*p* = 0.160 and *p* = 0.245, respectively). Therefore, the present findings indicate complementary prognostic contributions from comorbidity burden, frailty, and systemic inflammation but do not demonstrate unequivocal superiority of the three-domain model over all two-predictor combinations. These results demonstrate incremental prognostic performance rather than established clinical utility. Whether the observed improvement is sufficient to influence clinical decision-making requires external validation and assessment of clinical net benefit in independent cohorts.

Several limitations should be considered. First, the single-center design may restrict the applicability of the findings to other settings. In addition, patients with acute infection or autoimmune disease were excluded to reduce major disease-related alterations in the neutrophil, lymphocyte, and platelet counts used to calculate SII. Because acute infectious and inflammatory conditions are common among older adults presenting to the emergency department, this may further limit the generalizability of our findings to an unselected geriatric ED population, particularly to patients presenting with acute infectious or inflammatory conditions. Moreover, recruitment was conducted during periods when study personnel were available rather than through continuous enrollment of all eligible patients. Because a comprehensive screening log was not maintained, the total number of potentially eligible patients and the numbers not enrolled for each specific reason, including missing or incomplete clinical data, could not be reliably determined. Therefore, the possibility of selection bias cannot be excluded, and the generalizability of the findings should be interpreted accordingly. Second, although patients were prospectively followed for 30-day mortality, the observational design precludes causal inferences between the evaluated parameters and clinical outcomes. Third, frailty was assessed in the ED using patient and/or proxy interviews to estimate the patient’s usual baseline functional status. Although all assessors used the same standard CFS descriptors, the acute clinical context may have influenced recall or assessment, and formal assessor training and inter-rater reliability testing were not performed; therefore, some inter-rater variability cannot be excluded. Fourth, SII was derived from a single set of laboratory measurements obtained during the initial ED evaluation and may be influenced by transient factors such as acute physiological changes, hydration status, and medication use. In addition, a single measurement does not reflect dynamic changes in systemic inflammation during the course of illness. Fifth, we did not evaluate established emergency severity scores, such as NEWS2 or qSOFA; therefore, we could not determine whether the integrated model provides incremental prognostic value over existing emergency department risk assessment tools. Sixth, although the integrated prognostic model underwent internal validation using bootstrap resampling and demonstrated acceptable calibration, external validation in independent cohorts is required before its routine clinical application. The primary model was intentionally limited to three predictor parameters in view of the 60 available mortality events; nevertheless, the event count may have limited the precision of model estimates and the ability to reliably evaluate interaction effects or perform detailed subgroup analyses. Finally, this study evaluated 30-day all-cause mortality without systematic adjudication of cause-specific mortality; therefore, the prognostic value of the integrated model for specific causes of death and longer-term outcomes remains to be determined.

## 5. Conclusions

In the primary analysis, independent associations with 30-day mortality were observed for each of the three evaluated domains: comorbidity burden, frailty, and systemic inflammation. CCI had the highest AUC among the individual predictors, whereas the combined CCI, CFS, and ln(SII) model yielded the highest observed discrimination and acceptable internal calibration. However, its discriminative performance was not significantly greater than that of some two-predictor models, indicating that the incremental benefit of including all three domains was modest. These findings suggest that comorbidity burden, frailty, and systemic inflammation provide complementary prognostic information, while further studies are needed to determine whether their combined assessment offers clinically meaningful incremental value over simpler models. The generalizability and potential clinical applicability of the integrated model should be evaluated in larger, independent multicenter cohorts.

## Figures and Tables

**Table 1:** Demographic and clinical characteristics of the study population according to 30-day mortality.

Characteristics	Overall (*n* = 326)	Mortality (*n* = 60)	Survivors (*n* = 266)	*p* Value
Sex (male), *n* (%)	181 (55.5)	43 (71.7)	138 (51.9)	0.005
Age (years), median (IQR)	75 (70–82)	75 (72–82)	75 (70–81)	0.328
Height (cm), median (IQR)	167 (160–174)	170 (162–175)	165 (160–174)	0.075
Weight (kg), median (IQR)	79 (69–90)	75 (65–81)	80 (70–92)	0.002
Waist circumference (cm), mean ± SD	106 ± 18	97 ± 20	108 ± 17	<0.001
Comorbidities				
Hypertension, *n* (%)	244 (74.8)	40 (66.7)	204 (76.7)	0.106
Diabetes, *n* (%)	126 (38.7)	19 (31.7)	107 (40.2)	0.219
Coronary artery disease, *n* (%)	85 (26.1)	12 (20.0)	73 (27.4)	0.236
Congestive heart failure, *n* (%)	79 (24.2)	16 (26.7)	63 (23.7)	0.626
Chronic kidney disease, *n* (%)	71 (21.8)	16 (26.7)	55 (20.7)	0.310
Cerebrovascular disease, *n* (%)	40 (12.3)	14 (23.3)	26 (9.8)	0.004
Liver disease, *n* (%)	20 (6.1)	7 (11.7)	13 (4.9)	0.069
Peptic ulcer, *n* (%)	25 (7.7)	4 (6.7)	21 (7.9)	0.747
Peripheral vascular disease, *n* (%)	26 (8.0)	4 (6.7)	22 (8.3)	0.679
Chronic lung disease, *n* (%)	46 (14.1)	13 (21.7)	33 (12.4)	0.068
Malignancy, *n* (%)	62 (19.0)	29 (48.3)	33 (12.4)	<0.001
Smoking status				
Never smoked, *n* (%)	152 (46.6)	22 (36.7)	130 (48.9)	
Former smoker, *n* (%)	134 (41.1)	31 (51.7)	103 (38.7)	0.167
Current smoker, *n* (%)	40 (12.3)	7 (11.7)	33 (12.4)	
Physical activity				
<2 days/week, *n* (%)	266 (81.6)	54 (90.0)	212 (79.7)	0.063
2 days/week or more, *n* (%)	60 (18.4)	6 (10.0)	54 (20.3)	
Immunosuppressive medication use, *n* (%)	39 (12.0)	17 (28.3)	22 (8.3)	<0.001

IQR: Interquartile Range (25p, 75p); SD: standard deviation.

**Table 2:** Laboratory parameters and primary study variables according to 30-day mortality.

Laboratory Parameters	Overall (*n* = 326)	Mortality (*n* = 60)	Survivors (*n* = 266)	*p* Value
Leukocyte (10^3^/μL), median (IQR)	8.35 (6.41–11.00)	9.15 (6.75–12.68)	8.20 (6.41–10.68)	0.032
Lymphocyte (10^3^/μL), median (IQR)	1.40 (0.90–1.90)	1.20 (0.90–1.63)	1.42 (0.91–2.00)	0.094
Neutrophil (10^3^/μL), median (IQR)	6.00 (4.00–8.50)	7.45 (4.90–10.25)	5.80 (3.88–8.05)	0.001
Monocyte (10^3^/μL), median (IQR)	0.50 (0.40–0.70)	0.50 (0.32–0.70)	0.50 (0.40–0.70)	0.986
Hemoglobin (g/dL), median (IQR)	12.0 (10.4–13.6)	10.6 (9.28–12.2)	12.4 (10.9–13.8)	<0.001
Platelet (10^3^/μL), median (IQR)	226 (180–285)	257 (202–320)	223 (173–281)	0.003
Glucose (mg/dL), median (IQR)	124 (103–160)	126 (112–157)	123 (101–161)	0.458
Urea (mg/dL), median (IQR)	52 (38–74)	58 (38–100)	52 (37–71)	0.082
Creatinine (mg/dL), median (IQR)	1.05 (0.80–1.50)	1.08 (0.80–1.57)	1.04 (0.80–1.45)	0.578
eGFR (mL/min/1.73 m^2^), median (IQR)	58 (39–78)	60 (34–84)	57 (40–76)	0.667
Albumin (g/dL), median (IQR)	37 (34–41)	33 (30–36)	38 (35–41)	<0.001
ALT (U/L), median (IQR)	15 (11–22)	18 (11–24)	15 (11–22)	0.442
AST (U/L), median (IQR)	21 (16–28)	22 (17–31)	20 (16–28)	0.372
CRP (mg/L), median (IQR)	11.4 (4.50–56.3)	35.0 (10.0–97.8)	9.30 (3.92–42.8)	<0.001
Variables				
SII, median (IQR)	885 (513–1768)	1520 (829–2841)	794 (460–1513)	<0.001
CFS, median (IQR)	4 (3–5)	5 (4–7)	4 (3–5)	<0.001
CCI, median (IQR)	5 (4–7)	7 (5–9)	5 (4–6)	<0.001

IQR: Interquartile Range (25p, 75p), eGFR: estimated Glomerular Filtration Rate, ALT: Alanine Aminotransferase, AST: Aspartate Aminotransferase, CRP: C-reactive protein, SII: Systemic Immune-Inflammation Index, CFS: Clinical Frailty Scale, and CCI: Age-adjusted Charlson Comorbidity Index.

**Table 3:** Univariable and Multivariable Logistic Regression Analyses of CCI, CFS, and ln(SII) for 30-Day Mortality.

Variable	Univariable OR (95% CI)	*p* Value	Adjusted OR (95% CI)	*p* Value
CCI, per 1-point increase	1.435 (1.263–1.631)	<0.001	1.320 (1.145–1.520)	<0.001
CFS, per 1-point increase	1.546 (1.301–1.837)	<0.001	1.244 (1.022–1.515)	0.030
ln(SII)	1.783 (1.330–2.389)	<0.001	1.500 (1.096–2.053)	0.011

CCI: Age-adjusted Charlson Comorbidity Index; CFS: Clinical Frailty Scale; SII: Systemic Immune-Inflammation Index; OR: odds ratio; CI: confidence interval. Adjusted estimates were obtained from the integrated multivariable logistic regression model including CCI, CFS, and ln(SII).

**Table 4:** Diagnostic Performance of SII, CFS, and CCI for Predicting 30-Day Mortality.

Model	AUC	95% CI	Brier Score
CCI	0.741	0.676–0.800	0.133
CFS	0.703	0.636–0.768	0.137
ln(SII)	0.662	0.582–0.741	0.142
CCI + CFS	0.772	0.714–0.828	0.129
CCI + ln(SII)	0.777	0.720–0.835	0.129
CFS + ln(SII)	0.734	0.675–0.805	0.134
CCI + CFS + ln(SII)	0.793	0.741–0.851	0.127

## Data Availability

The data presented in this study are available on reasonable request from the corresponding author. The data are not publicly available due to privacy and ethical restrictions related to patient confidentiality.

## Supplementary Materials

The following supporting information can be downloaded at: https://www.mdpi.com/article/10.3390/jcm15186954/s1, Table S1: Sensitivity analysis excluding patients with malignancy. Multivariable logistic regression analysis for 30-day mortality; Table S2: Sensitivity analysis of the integrated model additionally adjusted for sex for 30-day mortality; Table S3: Sensitivity analysis additionally adjusting for immunosuppressive medication use.

## Abbreviations

The following abbreviations are used in this manuscript:

ED	Emergency Department
CCI	Age-adjusted Charlson Comorbidity Index
CFS	Clinical Frailty Scale
SII	Systemic Immune-Inflammation Index
ROC	Receiver Operating Characteristic
eGFR	Estimated Glomerular Filtration Rate
ALT	Alanine Aminotransferase
AST	Aspartate Aminotransferase
CRP	C-Reactive Protein
NEWS2	The National Early Warning Score 2
qSOFA	Quick Sequential Organ Failure Assessment

## References

[1] Shreya D., Fish P.N., Du D. (2025). Navigating the Future of Elderly Healthcare: A Comprehensive Analysis of Aging Populations and Mortality Trends Using National Inpatient Sample (NIS) Data (2010–2024). Cureus.

[2] Liu X., Yang X. (2024). Research Progress on Frailty in Elderly People. Clin. Interv. Aging.

[3] Charlson M.E., Pompei P., Ales K.L., MacKenzie C.R. (1987). A new method of classifying prognostic comorbidity in longitudinal studies: Development and validation. J. Chronic Dis..

[4] Ohta R., Ryu Y., Sano C., Ichinose K. (2025). Integrating Multimorbidity Assessment into Rheumatology Care: Prognostic Role of the Charlson Comorbidity Index in Systemic Lupus Erythematosus. Healthcare.

[5] Zhang N., Chen S., Shi Y., Wang Z., Jia R., Dai G. (2025). Charlson syndrome index predicted survival in pancreatic cancer patients received immunotherapy. Front. Immunol..

[6] Cornic A., Caraman D., Briere O., Gautier J., Annweiler C., Bourgeais A. (2026). BACK-UPUG study group. Frailty and inflammation predict prolonged stay in post-emergency geriatric units: A retrospective cohort study. J. Nutr. Health Aging.

[7] Park B., Alani Z., Sulistio E., Barazanchi A.W.H., Koea J., Vandal A., Hill A.G., MacCormick A.D. (2024). Frailty using the Clinical Frailty Scale to predict short- and long-term adverse outcomes following emergency laparotomy: Meta-analysis. BJS Open.

[8] Karpuzoglu E., Holladay S.D., Gogal R.M. (2025). Inflammaging: Triggers, molecular mechanisms, immunological consequences, sex differences, and cutaneous manifestations. Front. Immunol..

[9] Zhu Q., Dai L. (2025). Prognostic implications of systemic immune-inflammation index and systemic inflammation response index in hemodialysis patients. BMC Nephrol..

[10] Uyar S., Koca N., Oral A., Zorlu Görgülügil G. (2024). Impact of Obesity Severity on Hepatic Steatosis and Systemic Inflammatory Markers: A Comparative Analysis Across Obesity Classes. DAHUDER Med. J..

[11] Shiffer D., Reggiani F., Voza A. (2026). Frailty assessment tools in the emergency department and their predictive value for clinical outcomes: Review of the literature. Geroscience.

[12] Yılmaz A., Çon E. (2025). The Impact of Combining CIRS-G and Clinical Frailty Score on One-Month Mortality in Acute Coronary Syndrome. Healthcare.

[13] Haimovich A.D., Shah M.N., Southerland L.T., Hwang U., Patterson B.W. (2024). Automating risk stratification for geriatric syndromes in the emergency department. J. Am. Geriatr. Soc..

[14] Charlson M., Szatrowski T.P., Peterson J., Gold J. (1994). Validation of a combined comorbidity index. J. Clin. Epidemiol..

[15] Rockwood K., Song X., MacKnight C., Bergman H., Hogan D.B., McDowell I., Mitnitski A. (2005). A global clinical measure of fitness and frailty in elderly people. CMAJ.

[16] Özsürekci C., Balcı C., Kızılarslanoğlu M.C., Çalışkan H., Tuna Doğrul R., Ayçiçek G.Ş., Sümer F., Karabulut E., Yavuz B.B., Cankurtaran M. (2020). An important problem in an aging country: Identifying the frailty via 9 Point Clinical Frailty Scale. Acta Clin. Belg..

[17] Kaeppeli T., Rueegg M., Dreher-Hummel T., Brabrand M., Kabell-Nissen S., Carpenter C.R., Bingisser R., Nickel C.H. (2020). Validation of the Clinical Frailty Scale for Prediction of Thirty-Day Mortality in the Emergency Department. Ann. Emerg. Med..

[18] Hu B., Yang X.R., Xu Y., Sun Y.F., Sun C., Guo W., Zhang X., Wang W.M., Qiu S.J., Zhou J. (2014). Systemic immune-inflammation index predicts prognosis of patients after curative resection for hepatocellular carcinoma. Clin. Cancer Res..

[19] Anagnostou D., Theodorakis N., Kalantzi S., Spyridaki A., Chitas C., Milionis V., Kollia Z., Christodoulou M., Nella I., Spathara A. (2025). Predictive Value of Frailty, Comorbidity, and Patient-Reported Measures for Hospitalization or Death in Older Outpatients: Quality of Life and Depression as Prognostic Red Flags. Diagnostics.

[20] de Haan E., van Oosten B., van Rijckevorsel V.A.J.I.M., Kuijper T.M., de Jong L., Roukema G.R. (2024). Validation of the Charlson Comorbidity Index for the prediction of 30-day and 1-year mortality among patients who underwent hip fracture surgery. Perioper. Med..

[21] Westerberg M., Garmo H., Ludvigsson J.F., Stattin P., Gedeborg R. (2025). Discriminative Ability of the Charlson Comorbidity Index for Long-Term Mortality in a General Population: Nationwide, Population-Based Study of 10 Million Adults in Sweden. Clin. Epidemiol..

[22] Riviati N., Legiran, Indrajaya T., Saleh I., Ali Z., Irfannuddin, Probosuseno I.B. (2024). Serum Albumin as Prognostic Marker for Older Adults in Hospital and Community Settings. Gerontol. Geriatr. Med..

[23] Li Z.Q., Liu X.X., Wang X.F., Shen C., Cao F., Guan X.M., Zhang Y., Liu J.P. (2024). Synergistic impact of plasma albumin and cognitive function on all-cause mortality in Chinese older adults: A prospective cohort study. Front. Nutr..

[24] Hong S.N., Lai F.T.T., Wang B., Choi E.P.H., Wong I.C.K., Lam C.L.K., Wan E.Y.F. (2024). Age-specific multimorbidity patterns and burden on all-cause mortality and public direct medical expenditure: A retrospective cohort study. J. Epidemiol. Glob. Health.

[25] Damiano C., Costanzo S., Marcozzi B., Panzera T., Donfrancesco C., Di Castelnuovo A., Lo Noce C., Magnacca S., Triolo F., Zazzara M.B. (2025). BIO-SIGN Study Investigators. Multimorbidity patterns and mortality in older adults: A two-cohort pooled analysis. Aging Clin. Exp. Res..

[26] Javadzadeh D., Karlson B.W., Alfredsson J., Ekerstad E., Hellberg J., Herlitz J., Ekerstad N. (2024). Clinical Frailty Scale score is a predictor of short-, mid- and long-term mortality in critically ill older adults (≥70 years) admitted to the emergency department: An observational study. BMC Geriatr..

[27] Chung H.S., Choi Y., Lim J.Y., Kim K., Choi Y.H., Lee D.H., Bae S.J. (2025). The clinical frailty scale improves risk prediction in older emergency department patients: A comparison with qSOFA, NEWS2, and REMS. Sci. Rep..

[28] Zhou L., Huang C., Zhu X., Ma Z. (2024). Combined Systemic Immune-inflammatory Index (SII) and Geriatric Nutritional Risk Index (GNRI) predict survival in elderly patients with hip fractures: A retrospective study. J. Orthop. Surg. Res..

[29] Durgun H.M., Özkul E., Yaman M., Şen A. (2026). Prognostic Value of HALP Score, PNI, and SII in Predicting 1-Year Mortality in Geriatric Femoral Fractures: A 5-Year Emergency Department Cohort Study. Med. Sci. Monit..

