# Supplementary material for: Integrated Risk Stratification Using Comorbidity Burden, Frailty, and Systemic Inflammation to Predict 30-Day Mortality in Older Emergency Department Patients: A Prospective Cohort Study
Source: J Clin Med. 2026 Sep 8;15(18):6954. doi: 10.3390/jcm15186954 (PMC13607925; doi:10.3390/jcm15186954)
Supplement: Supplementary file 1 [file jcm-15-06954-s001.zip › jcm-4498110-supplementary.pdf]

Supplementary Table S1. Sensitivity analysis excluding patients with malignancy. Multivariable logistic regression analysis for 30-day mortality.

Variable	Adjusted OR	95% CI	p Value
CCI	1.313	1.061–1.626	0.012
CFS	1.241	0.956–1.611	0.105
ln(SII)	1.486	1.004–2.200	0.048

Supplementary Table S2. Sensitivity Analysis of the Integrated Model Additionally Adjusted for Sex for 30-Day Mortality

Variable	Adjusted OR	95% CI	p Value
CCI	1.303	1.127–1.507	**<0.001**
CFS	1.329	1.080–1.635	0.007
ln(SII)	1.423	1.033–1.961	0.031
Male Sex	2.906	1.459–5.788	0.002

Supplementary Table S3. Sensitivity analysis additionally adjusting for immunosuppressive medication use

Variable	Adjusted OR	95% CI	p Value
CCI	1.271	1.101–1.469	0.001
CFS	1.250	1.023–1.529	0.029
ln(SII)	1.492	1.087–2.048	0.013
Immunosuppressive medication use	2.948	1.315–6.609	0.009
